# Synthesis and antimicrobial evaluation of new 1,4-dihydro-4-pyrazolylpyridines and 4-pyrazolylpyridines

**DOI:** 10.1186/2191-2858-1-5

**Published:** 2011-08-03

**Authors:** Om Prakash, Khalid Hussain, Ravi Kumar, Deepak Wadhwa, Chetan Sharma, Kamal R Aneja

**Affiliations:** 1 Institute of Pharmaceutical Sciences, Kurukshetra University, Kurukshetra 136 119, India; 2 Department of Chemistry, Guru Nanak Khalsa College, Yamunanagar 135001, India; 3 Department of Chemistry, Dyal Singh College, Karnal 132 001, India; 4 Department of Chemistry, Kurukshetra University, Kurukshetra 136 119, India; 5 Department of Microbiology, Kurukshetra University, Kurukshetra 136 119, India

**Keywords:** 1,4-Dihydro-4-pyrazolylpyridines, 4-pyrazolylpyridines, HTIB, oxidation, antibacterial activity, antifungal activity

## Abstract

**Background:**

Dialkyl 1,4-dihydro-2,6-dimethylpyridine-3,5-dicarboxylates (1,4-DHP) have now been recognized as vital drugs. Some of these derivatives such as amlodipine, felodipine, isradipine, etc. have been commercialized. In view of wide range of biological properties associated with 1,4-DHP and owing to the biological importance of the oxidation step of 1,4-DHP, we carried out the synthesis and antimicrobial evaluation of new diethyl 1,4-dihydro-2,6-dimethyl-4-(3-aryl-1-phenyl-4-pyrazolyl)pyridine-3,5-dicarboxylates (**2a-g**) and diethyl 2,6-dimethyl-4-(3-aryl-1-phenyl-4-pyrazolyl)pyridine-3,5-dicarboxylates (**3a-g**).

**Results:**

Synthesis of a series of new diethyl 1,4-dihydro-2,6-dimethyl-4-(3-aryl-1-phenyl-4-pyrazolyl)pyridine-3,5-dicarboxylates (**2a-g**) has been accomplished by multicomponent cyclocondensation reaction of ethyl acetoacetate, 3-aryl-1-phenyl pyrazole-4-carboxaldehyde (**1a-g**) and ammonium acetate. The dihydropyridines **2a-g **were smoothly converted to new diethyl 2,6-dimethyl-4-(3-aryl-1-phenyl-4-pyrazolyl)pyridine-3,5-dicarboxylates (**3a-g**) using HTIB ([Hydroxy (tosyloxy)iodo]benzene, Koser's reagent) as the oxidizing agent. The antimicrobial studies of the title compounds, **2a-g **&**3a-g**, are also described.

**Graphical abstract:**

Synthesis of a series of new diethyl 1,4-dihydro-2,6-dimethyl-4-(3-aryl-1-phenyl-4-pyrazolyl)pyridine-3,5-dicarboxylates (**2a-g**), their aromatization using HTIB ([Hydroxy(tosyloxy)iodo]benzene, Koser's reagent) to afford new diethyl 2,6-dimethyl-4-(3-aryl-1-phenyl-4-pyrazolyl)pyridine-3,5-dicarboxylates (**3a-g**), and antimicrobial studies of **2a-g **and **3a-g **are reported.

## Background

**Figure 1:**
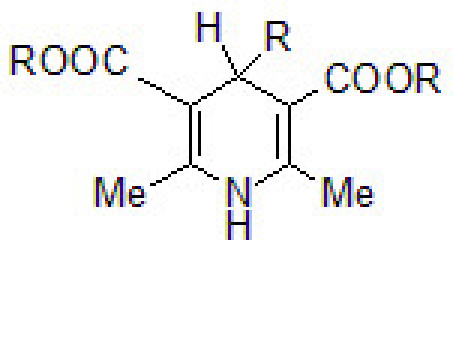
**1,4-DHP**.

Dialkyl 1,4-dihydro-2,6-dimethylpyridine-3,5-dicarboxylates (1,4-DHP; Figure 1) have now been recognized as vital drugs. Some of these derivatives, such as amlodipine, felodipine, isradipine, etc. have been commercialized, and it has been proven that their therapeutic success is related to their efficacy to bind to calcium channels and consequently to decrease the passage of the transmembrane calcium current [1-3]. Further, cerebrocrast, a dihydropyridine derivative, has been introduced as a neuroprotective agent [4]. Together with calcium channel blocker and neuroprotective activity, a number of dihydropyridine derivatives have been found as vasodilators, antihypertensive, bronchodilators, antiatherosclerotic, hepatoprotective, antitumour, antimutagenic, geroprotective, antidiabetic and antiplatelet aggregation agents [5-9]. In a recent article, 4-[5-chloro-3-methyl-1-phenyl-1H-pyrazol-4-yl]-dihydropyridines have been shown to possess significant antimicrobial activity [10].

In addition to above, aromatization of 1,4-DHP has also attracted considerable attention in recent years as Böcker has demonstrated that metabolism of the above drugs involves a cytochrome P-450 catalysed oxidation in the liver [11].

In view of wide range of biological properties associated with 1,4-DHP and the biological importance of the oxidation step of 1,4-DHP, we carried out the synthesis and antimicrobial evaluation of new diethyl 1,4-dihydro-2,6-dimethyl-4-(3-aryl-1-phenyl-4-pyrazolyl)pyridine-3,5-dicarboxylates (**2a-g**) and diethyl 2,6-dimethyl-4-(3-aryl-1-phenyl-4-pyrazolyl)pyridine-3,5-dicarboxylates (**3a-g**).

## Results and discussion

### Chemistry

**Scheme 1:**
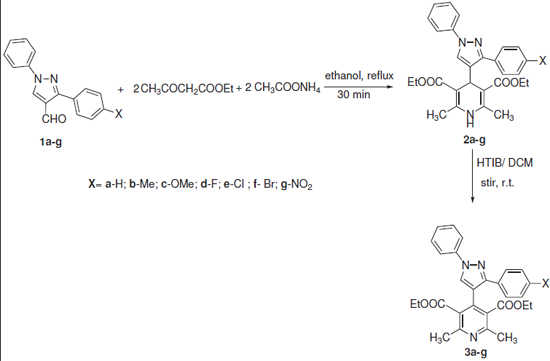
**Synthesis of 1,4-DHP (2) and aromatization of 2 to 3 using HTIB**.

The synthetic scheme used for the synthesis of diethyl 1,4-dihydro-2,6-dimethyl-4-(3-aryl-1-phenyl-4-pyrazolyl) pyridine-3,5-dicarboxylates (**2a-g**) is outlined in Scheme 1. Synthesis of the title compounds **2a-g **was accomplished by multicomponent cyclocondensation reaction of ethyl acetoacetate, 3-aryl-1-phenyl-pyrazole-carboxaldehyde (**1a-g**) and ammonium acetate in ethanol. The purity of the compounds was checked by TLC and elemental analysis. Spectral data (IR, ^1^H NMR (see additional files 1, 2, 3, 4 and 5, mass) of the newly synthesized compounds **2a-g **were in full agreement with their proposed structures. The IR spectra of compounds **2a-g **exhibited characteristic peak at approximately 1697 cm^-1 ^because of the presence of ester group (-COOEt), and peak due to -N-H stretch appeared in the region 3300-3317 cm^-1^. In ^1^H NMR of compounds **2a-g**, the protons of C_4_-H and -NH of the dihydropyridine ring resonate between δ 5 and 6 ppm.

Hypervalent iodine (III) and iodine (V) reagents have been used as green-oxidants for a variety of substrates [12-17]. Amongst the various reagents used, HTIB has been reported to serve as a mild, fast and efficient oxidant for the aromatization of Hantzsch 1,4-dihydropyridines to pyridines [18].

Thus, diethyl 1,4-dihydro-2,6-dimethyl-4-(3-aryl-1-phenyl-4-pyrazolyl) pyridine-3,5-dicarboxylates (**2a-g**) were further oxidized by treating with HTIB (Koser's reagent) in dichloromethane (CH_2_Cl_2_) at room temperature to afford new diethyl 2,6-dimethyl-4-(3-aryl-1-phenyl-4-pyrazolyl) pyridine-3,5-dicarboxylates (**3a-g**) in good-to-excellent yields (Scheme 1). All the compounds **3a-g **were unambiguously characterized on the basis of their spectral (IR, ^1^H NMR (see additional files 6, 7, 8, 9, 10, 11 and 12) and mass) and elemental data.

**Scheme 2:**
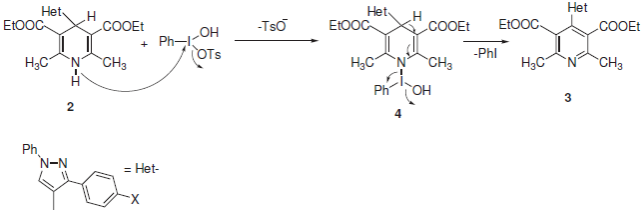
**Proposed mechanism for the oxidation of 2 to 3**.

A plausible mechanism for the oxidation of dihydropyridines **2 **to **3 **is outlined in Scheme 2. The probable mechanism might involve the attack by N-H on PhI(OH)OTs, leading to the formation of intermediate **4**. The intermediate **4 **finally loses a molecule of iodobenzene (PhI) to give **3**.

### Pharmacology

All the synthesized compounds, **2a-g **and **3a-g**, were evaluated *in vitro *for their antibacterial activity against two gram-positive bacterial strains, *Staphylococcus aureus *&*Bacillus subtilis *and two gram-negative bacteria, namely, *Escherichia coli *and *Pseudomonas aeruginosa *and their activities were compared with a well-known commercial antibiotic, ciprofloxacin. In addition, the synthesized compounds were also evaluated for their antifungal activity against *Aspergillus niger *&*Aspergillus flavus *and their antifungal potential was compared to reference drug, fluconazole. Compounds possessed variable antibacterial activities against Gram-positive bacteria, *S. aureus*, *B. subtilis*. However, the compounds in this series were not effective against any Gram-negative bacteria, neither against *E. coli *nor against *P. aeruginosa*. Results of antibacterial evaluation are summarized in Table 1.

**Table 1:** Antibacterial activity of chemical compounds through agar well diffusion method

Compound	Diameter of growth of inhibition zone (mm)^a^			
	
	*S. aureus*	*Bacillus Subtilis*	*E. coli*	*P. aeruginosa*
**2a**	15.6	16.3	-	-
**2b**	18.6	15.6	-	-
**2c**	16.3	15.6	-	-
**2d**	17.6	16.3	-	-
**2e**	16	15.3	-	-
**2f**	15.6	14	-	-
**2g**	15.3	16.6	-	-
**3a**	20	19.3	-	-
**3b**	15	15.6	-	-
**3c**	15.3	16.6	-	-
**3d**	16.6	14.6	-	-
**3e**	16.6	18.3	-	-
**3f**	15.3	16.6	-	-
**3g**	16.3	18.6	-	-
Ciprofloxacin	27.6	26.3	25.0	25.3

-, No activity

^a^Values, including diameter of the well (8 mm), are means of three replicates

Compounds **2a-g **and **3a-g **showed zones of inhibition ranging between 14 and 20 mm. On the basis of the zones of inhibition produced against the test bacteria, compounds **2b **and **3a **were found to be most effective against *S. aureus*, showing the maximum zones of inhibition at 18 and 20 mm, respectively, and compounds **3a**, **3e **and **3g **were found to be most effective against *B. subtilis*. The remaining compounds showed fair activity against gram-positive bacterial strains (Table 1). In the whole series, the MIC (minimum inhibitoty concentration) values of various tested chemical compounds ranged between 64 and 256 μg/mL against gram-positive bacteria. Compounds **2b **and **3a **displayed good antibacterial activity with the lowest MIC value, 64 μg/ml against *S. aureus*. Three compounds, **3a**, **3e **and **3g **possessed antibacterial activity with MIC value of 64 μg/mL against *B. subtilis *(Table 2).

**Table 2:** MIC (in μg/mL) of compounds obtained using macrodilution method

Compound	*S. aureus*	*Bacillus Subtilis*	Compound	*S. aureus*	*Bacillus* *Subtilis*
**2a**	128	128	**3a**	64	64
**2b**	64	128	**3b**	128	128
**2c**	128	128	**3c**	128	128
**2d**	128	128	**3d**	128	256
**2e**	128	128	**3e**	128	64
**2f**	128	256	**3f**	128	128
**2g**	128	128	**3g**	128	64
Ciprofloxacin	5	5			

Amongst the synthesized compounds, six compounds **2a**, **2d**, 2**g**, 3**a**, **3c **and **3d **showed more than 50% mycelial growth inhibition against ***A. niger ***whereas compounds, **2a**, **2e**, **2f**, **3a**, **3d **and **3f **were found to be active against *A. flavus *(Table 3).

**Table 3:** Antifungal activity of chemical compounds through poisoned food method (mycelial growth inhibition) (%)

Compound	*A. niger*	*A. flavus*	Compound	*A. niger*	*A. flavus*
**2a**	51.1	58.8	**3a**	52.5	51.1
**2b**	50	44.4	**3b**	48.8	45.5
**2c**	48.8	50	**3c**	51.1	50
**2d**	52.5	48.8	**3d**	55.5	52.5
**2e**	45.5	51.1	**3e**	45.5	44.4
**2f**	47.7	52.5	**3f**	50	51.1
**2g**	51.1	48.8	**3g**	48.8	44.4
Fluconazole	81.1	77.7			

From the overall result it is evident that compound **3a **could be identified as the most biologically active member within this study with good antifungal and antibacterial profile.

## Conclusions

A series of diethyl 1,4-dihydro-2,6-dimethyl-4-(3-aryl-1-phenyl-4-pyrazolyl)pyridine-3,5-dicarboxylates (**2a-g**) and diethyl 2,6-dimethyl-4-(3-aryl-1-phenyl-4-pyrazolyl)pyridine-3,5-dicarboxylates (**3a-g**) has been synthesized with the hope of discovering new structure leads. Compounds **2b **and **3a **were found to be most effective against *S. aureus *showing the maximum zones of inhibition of 18 and 20 mm, respectively, and compounds **3a**, **3e **and **3g **were found to be most effective against *B. subtilis*. Moreover, six compounds **2a**, **2d**, **2g**, **3a**, **3c **and **3d **showed more than 50% mycelial growth inhibition against *A. niger *whereas compounds, **2a**, **2e**, **2f**, **3a**, **3d **and **3f **were found to be active against *A. flavus*; however, no compound was found superior over the reference drug.

Finally, compound **3a **could be identified as the most biologically active member within this study with an interesting antibacterial and antifungal profile.

## Experimental

### Chemical synthesis

Melting points were taken in open capillaries and are uncorrected. IR spectra were recorded on Perkin-Elmer IR spectrophotometer. The ^1^H NMR spectra were recorded on Brucker 300 MHz instrument. The chemical shifts are expressed in ppm units downfield from an internal TMS standard. 3-Aryl-1-phenylpyrazole-4-carboxaldehydes (**1a-h**), needed for the present study, were synthesized by Vilsmeier-Haack reaction according to the literature procedure [19].

#### Synthesis of diethyl 1,4-dihydro-2,6-dimethyl-4-(3-aryl-1-phenyl-4-pyrazolyl) pyridine-3,5-dicarboxylates (2a-g)

General procedure: A mixture of appropriate 3-aryl-1-phenylpyrazole-4-carboxaldehyde (**1**, 10 mmol), ethyl acetoacetate (20 mmol) and ammonium acetate (22 mmol) in ethanol was allowed to reflux on water bath for 25-30 min. After completion of the reaction, the reaction mixture was cooled to room temperature to give pure diethyl 1,4-dihydro-2,6-dimethyl-4-(3-aryl-1-phenyl-4-pyrazolyl) pyridine-3,5-dicarboxylates (**2a-g**).

#### Characterization data of diethyl 1,4-dihydro-2,6-dimethyl-4-(3-aryl-1-phenyl-4-pyrazolyl) pyridine-3,5-dicarboxylates (2a-g)

**2a**: M.p.: 124°C; yield: 74%; IR (ν_max_, cm^-1^, KBr): 3323 (NH stretch), 1690 (-COOEt), 1207; ^1^H NMR (CDCl_3_, δ, ppm): 1.069-1.115 (t, 6H), 2.237 (s, 6H), 3.744-4.068 (m, 4 H), 5.318 (s, 1 H), 5.544 (s, 1 H), 7.221-7.424 (m, 4 H), 7.806 (s, 1 H), 7.681-7.868 (m, 6 H); mass: *m*/*z *472.30 (M^+ ^+ 1, 100%).

Anal. Calcd for C_28_H_29_N_3_O_4_: C 71.33, H 6.15, N 8.91; found: C 71.34, H 6.18, N 8.94; C 71.33, H 6.15, N 8.91.

**2b**: M.p.: 189°C; yield: 70%; IR (ν_max_, cm^-1^, KBr): 3325 (NH stretch), 1697 (-OOEt), 1643, 1211; ^1^HNMR (CDCl_3_, δ, ppm): 1.032-1.087 (t, 6 H), 2.225 (s, 6 H), 2.401 (s, 3 H), 3.730-4.095 (m, 4 H), 5.306 (s, 1 H), 5.722 (bs, 1 H), 7.205-7.282 (m, 3 H), 7.381-7.450 (m, 2 H), 7.664-7.692 (m, 2 H), 7.733 (s, 1 H), 7.742-7.769 (d, 2 H, *J *= 8.1 Hz); mass: *m*/*z *486.20 (M^+ ^+ 1, 100%).

Anal. Calcd for C_29_H_31_N_3_O_4_: C 71.75, H 6.39, N 8.66; found: C 71.71, H 6.42, N 8.66.

**2c**: M.p.: 139°C; yield: 78%; IR (ν_max_, cm^-1^, KBr): 3317 (NH stretch), 1697 (-COOEt), 1643, 1211; ^1^H NMR (CDCl_3_, δ, ppm): 1.079-1.127 (t, 6 H), 2.250 (s, 6 H), 3.866 (s, 3 H), 3.801-4.102 (m, 4 H), 5.288 (s, 1 H), 5.561 (s, 1 H), 6.962-6.991 (d, 2 H, *J *= 8.7 Hz), 7.209-7.440 (m, 3 H), 7.670-7.697 (d, 2 H, *J *= 8.7 Hz) 7.742 (s, 1 H), 7.785-7.814 (d, 2 H, *J *= 8.7 Hz); mass: *m*/*z *502.32 (M^+ ^+ 1, 100%).

Anal. Calcd for C_29_H_31_N_3_O_5_: C 69.46, H 6.19, N 8.38; found: C 69.42, H 6.24, N 8.37.

**2d**: M.p.: 175°C; yield: 72%; IR (ν_max_, cm^-1^, KBr): 3333 (NH stretch), 1697 (-COOEt), 1643, 1211; ^1^H NMR (CDCl_3_, δ, ppm): 0.940-0.975 (t, 6 H), 2.521 (s, 6 H), 4.102-4.132 (m, 4 H), 5.175 (s, 1 H), 5.562 (s, 1 H), 6.962-6.991 (d, 2 H, *J *= 8.7 Hz), 7.281-7.513 (m, 5 H), 7.734 (d, 2 H, *J *= 7.5 Hz), 7.922 (s, 1 H); mass: *m*/*z *490.26 (M^+ ^+ 1, 100%)

Anal. Calcd for C_28_H_28_N_3_O_4_F: C 68.71, H 5.73, N 8.58; found: C 68.72, H 5.75, N 8.56.

**2e**: M.p.: 185°C; Yield: 76%; IR (ν_max_, cm^-1^, KBr): 3317 (NH stretch), 1697 (-COOEt), 1636, 1211; ^1^H NMR (CDCl_3_, δ, ppm): 1.072-1.119 (t, 6 H), 2.280 (s, 6 H), 3.790-4.080 (m, 4 H), 5.285 (s, 1 H), 5.551 (s, 1 H), 7.235-7.454 (m, 5 H), 7.668-7.694 (d, 2 H) 7.814 (s, 1 H), 7.863-7.891 (d, 2 H, *J *= 8.4 Hz); mass: *m*/*z *506.26, 508.24.

Anal. Calcd for C_28_H_28_N_3_O_4_Cl: C 66.47, H 5.54, N 8.31; found: C 66.47, H 5.55, N 8.31.

**2f**: M.p.: 174°C; yield: 72%; IR (ν_max_, cm^-1^, KBr): 3564 (NH stretch), 1728 (-COOEt), 1242; ^1^H NMR (CDCl_3_, δ, ppm): 1.072-1.119 (t, 6 H), 2.275 (s, 6 H), 3.764-4.104 (m, 4 H), 5.284 (s, 1 H), 5.581 (s, 1 H), 7.234-7.452 (m, 3 H), 7.561-7.588 (d, 2 H, *J *= 7.8 Hz), 7.665-7.691 (d, 2 H, *J *= 7.8 Hz) 7.753 (s, 1 H), 7.806-7.834 (d, 2 H, *J *= 8.4 Hz); mass: *m*/*z *550.31, 552.31.

Anal. Calcd for C_28_H_28_N_3_O_4_Br: C 61.20, H 5.10, N 7.65; found: C 61.09, H 5.14, N 7.64.

**2g**: M.p.: 198°C; yield: 70%; IR (ν_max_, cm^-1^, KBr): 3302 (NH stretch), 1697 (-COOEt), 1636, 1211; ^1^H NMR (CDCl_3_, δ, ppm): 1.026-1.071 (t, 6 H), 2.325 (s, 6 H), 3.775-4.047 (m, 4 H), 5.335 (s, 1 H), 5.766 (s, 1 H), 7.282-7.473 (m, 4 H), 7.684-7.709 (d, 2 H, *J *= 7.5 Hz), 7.801 (s, 1 H), 8.254-8.344 (m, 3 H); mass: *m*/*z *517.29 (M^+ ^+ 1, 100%).

Anal. Calcd for C_28_H_28_N_4_O_6_: C 65.11, H 5.42, N 10.85; found: C 65.13, H 5.47, N 10.83.

#### Synthesis of diethyl 2,6-dimethyl-4-(3-aryl-1-phenyl-4-pyrazolyl)pyridine-3,5-dicarboxylates (3a-g)

General procedure: To a solution of appropriate 1,4-DHP (**2**, 10 mmol) in dichloromethane, was added HTIB (12 mmol) and the mixture was stirred at room temperature. The progress of the reaction was monitored by TLC. Reaction was completed in 4-5 min. After the completion of reaction, the reaction mixture was washed with aqueous NaHCO_3 _solution. Organic phase was then separated, dried and concentrated on water bath. Crude product, thus obtained, was purified by silica gel column chromatography using Pet ether/EtOAc (20:1) as eluent to afford pure diethyl 2,6-dimethyl-4-(3-aryl-1-phenyl-4-pyrazolyl)pyridine-3,5-dicarboxylates (**3a-g**).

#### Characterization data of dimethyl 2,6-dimethyl-4-pyrazolylpyridine-3,5-dicarb oxylates (3a-g)

**3a**: M.p.: 111°C; yield: 68%; IR (ν_max_, cm^-1^, KBr): 1736, 1233; ^1^H NMR (CDCl_3_, δ, ppm): 0.911-0.997 (t, 6 H), 2.613 (s, 6 H), 3.910-4.07 (m, 4 H), 7.110-7.313 (m, 4 H), 7.817 (s, 1 H), 7.581-7.690 (m, 6 H); mass: *m*/*z *470.20 (M^+ ^+ 1, 100%).

Anal. Calcd for C_28_H_27_N_3_O_4_: C 71.64, H 5.76, N 8.95; found: C 71.63, H 5.79, N 8.93.

**3b**: M.p.: 105°C; yield: 69%; IR (ν_max_, cm^-1^, KBr): 1720, 1234; ^1^H NMR (CDCl_3_, δ, ppm): 0.913-0.960 (t, 6 H), 2.611 (s, 6 H), 2.468 (s, 3H), 3.923-4.072 (q, 4 H), 6.839-6.868 (d, 2H, J=8.7 Hz), 7.280-7.501 (m, 5 H), 7.732-7.759 (d, 2 H, *J *= 8.7 Hz), 7.905 (s, 1 H); mass: *m*/*z *484.40 (M^+ ^+ 1, 100%).

Anal. Calcd for C_29_H_29_N_3_O_4_: C 72.05, H 6.00, N 8.70; found: C 72.06, H 6.05, N 8.70.

**3c**: M.p.: 136°C; Yield- 72%; IR (ν_max_, cm^-1^, KBr): 1740, 1034; ^1^H NMR (CDCl_3_, δ, ppm): 0.913-0.998 (t, 6 H), 2.612 (s, 6 H), 3.808 (s, 3 H), 3.924-4.08 (q, 4 H), 6.835-6.864 (d, 2 H, *J *= 8.7 Hz), 7.311-7.501 (m, 5 H), 7.732-7.759 (d, 2 H, *J *= 8.7 Hz), 7.905 (s, 1 H); mass: *m*/*z *500.29 (M^+ ^+ 1, 100%).

Anal. Calcd for C_29_H_29_N_3_O_5_: C 69.73, H 5.81, N 8.41; found: C 69.71, H 5.83, N 8.40.

**3d**: M.p.: 121°C; yield: 70%; IR (ν_max_, cm^-1^, KBr): 1728, 1236, 1037; ^1^H NMR (CDCl_3_, δ, ppm): 0.924-0.971 (t, 6 H), 2.615 (s, 6 H), 3.905-4.105 (q, 4 H), 6.987-7.044 (m, 2 H), 7.280-7.365 (m, 1 H), 7.469-7.622 (m, 4 H), 7.733-7.759 (d, 2 H, *J *= 7.8 Hz), 7.923 (s, 1 H); mass: *m*/*z *488.36 (M^+ ^+ 1, 100%).

Anal. Calcd for C_28_H_26_N_3_O_4_F: C 68.99, H 5.38, N 8.62; found: C 68.95, H 5.37, N 8.63.

**3e**: M.p.: 101-102°C, lit [20] M.p.: 101-102°C; Yield: 65%.

**3f**: M.p.: 115°C; yield: 70%; IR (ν_max_, cm^-1^, KBr): 1734, 1030; ^1^H NMR (CDCl_3_, δ, ppm): 0.940-0.962 (t, 6 H), 2.617 (s, 6 H), 3.957-4.039 (q, 4 H), 7.200-7.495 (m, 7 H), 7.732-7.756 (d, 2 H, *J *= 7.2 Hz), 7.921 (s, 1 H); mass: *m*/*z *548.20, 550.20.

Anal. Calcd for C_28_H_26_N_3_O_4_Br: C 61.42, H 4.75, N 7.68; found: C 61.31, H 4.79, N 7.69.

**3g**: M.p.: 172°C; yield: 68%; IR (ν_max_, cm^-1^, KBr): 1728, 1234, 1034; ^1^H NMR (CDCl_3_, δ, ppm): 0.895-0.941 (t, 6 H), 2.632 (s, 6 H), 3.923-4.039 (m, 4 H), 7.279-7.410 (m, 3 H), 7.499-7.769 (m, 4 H), 7.960 (s, 1 H), 8.178-8.207 (d, 2 H, *J *= 7.5 Hz); mass: *m*/*z *515.26 (M^+ ^+ 1, 100%).

Anal. Calcd for C_28_H_26_N_4_O_6_: C 64.37, H 4.98, N 10.73; found: C 65.34, H 5.08, N 10.87.

## Pharmacology

### Test microorganisms

Total six microbial strains were selected on the basis of their clinical importance in causing diseases in humans. Two Gram-positive bacteria (*S. aureus *MTCC 96 and *B. subtilis *MTCC 121); two Gram-negative bacteria (*E. coli *MTCC 1652 and *P. aeruginosa *MTCC 741) and two fungi (*A. niger *and *A. flavus*) the ear pathogens isolated from the patients of Kurukshetra [21], were used in the present study for the evaluation of antimicrobial activities of the chemical compounds. All the cultures were procured from Microbial Type Culture Collection (MTCC), IMTECH, Chandigarh. The bacteria and fungi were subcultured on Nutrient agar and Sabouraud's dextrose agar (SDA), respectively, and incubated aerobically at 37°C.

### In vitro antibacterial activity

The antibacterial activities of compounds, **2a-g **and **3a-g**, were evaluated by the agar well diffusion method. All the cultures were adjusted to 0.5 McFarland standard, which is visually comparable to a microbial suspension of approximately 1.5 × 10^8 ^cfu/mL. 20 mL of Mueller Hinton agar medium was poured into each Petri plate, and the agar plates were swabbed with 100 μL inocula of each test bacterium and kept for 15 min for adsorption. Using sterile cork borer of 8-mm diameter, wells were bored into the seeded agar plates, and these were then loaded with a 100 μL volume with concentration of 2.0 mg/mL of each compound reconstituted in the dimethylsulphoxide (DMSO). All the plates were incubated at 37°C for 24 h. Antibacterial activity of each compound was evaluated by measuring the zone of growth inhibition against the test organisms with zone reader (Hi Antibiotic zone scale). DMSO was used as a negative control whereas ciprofloxacin was used as a positive control. This procedure was performed in three replicate plates for each organism [22,23].

### Determination of minimum inhibitory concentration

Minimum inhibitory concentration (MIC) is the lowest concentration of an antimicrobial compound that will inhibit the visible growth of a microorganism after overnight incubation. MIC of the compounds against bacterial strains was tested through a macrodilution tube method as recommended by NCCLS [24]. In this method, various test concentrations of chemically synthesized compounds were made from 256 to 1 μg/mL in sterile tubes, 1-10. 100 μL sterile Mueller Hinton Broth was poured in each sterile tube, and followed by addition of 200 μL test compound in tube 1. Twofold serial dilutions were carried out from tubes 1 to 10, and excess broth (100 μL) was discarded from the tube 10. To each tube, 100 μL of standard inoculum (1.5 × 10^8 ^cfu/mL) was added. Ciprofloxacin was used as control. Turbidity was observed after incubating the inoculated tubes at 37°C for 24 h.

### In vitro antifungal activity

The antifungal activity of the synthesized chemical compounds was evaluated by poison food technique. The moulds were grown on SDA at 25°C for 7 days and used as inocula. 15 mL of molten SDA (45°C) was poisoned by the addition of 100 μL volume of each compound having concentration of 4.0 mg/mL, reconstituted in the DMSO, poured into a sterile Petri plate and allowed to solidify at room temperature. The solidified poisoned agar plates were inoculated at the centre with fungal plugs (8-mm diameter), obtained from the actively growing colony and incubated at 25°C for 7 days. DMSO was used as the negative control whereas fluconazole was used as the positive control. The experiments were performed in triplicates. Diameter of the fungal colonies was measured and expressed as percent mycelial inhibition determined by applying the following formula [25]:

where *dc *is the average diameter of fungal colony in negative control plates, and *dt *the average diameter of fungal colony in experimental plates.

## Abbreviations

1,4-DHP: dialkyl 1,4-dihydro-2,6-dimethylpyridine-3,5-dicarboxylates; DMSO: dimethylsulphoxide; HTIB: hydroxy (tosyloxy)iodobenzene; MIC: minimum inhibitory concentration; MTCC: microbial type culture collection; SDA: Sabouraud dextrose agar.

## Competing interests

The authors declare that they have no competing interests.

## Supplementary Material

Additional file 1 **1HNMR spectrum of compound 2b**. Click here for file

Additional file 2 **1HNMR spectrum of compound 2c**. Click here for file

Additional file 3 **1HNMR spectrum of compound 2e**. Click here for file

Additional file 4 **1HNMR spectrum of compound 2f**. Click here for file

Additional file 5 **1HNMR spectrum of compound 2g**. Click here for file

Additional file 6 **1HNMR spectrum of compound 3a**. Click here for file

Additional file 7 **1HNMR spectrum of compound 3b**. Click here for file

Additional file 8 **1HNMR spectrum of compound 3c**. Click here for file

Additional file 9 **1HNMR spectrum of compound 3d**. Click here for file

Additional file 10 **1HNMR spectrum of compound 3e**. Click here for file

Additional file 11 **1HNMR spectrum of compound 3f**. Click here for file

Additional file 12 **1HNMR spectrum of compound 3g**. Click here for file
