# Supplementary figures and images for: Synthesis and antimicrobial evaluation of new 1,4-dihydro-4-pyrazolylpyridines and 4-pyrazolylpyridines
Source: Org Med Chem Lett. 2011 Aug 3;1:5. doi: 10.1186/2191-2858-1-5 (PMC3279143; doi:10.1186/2191-2858-1-5)

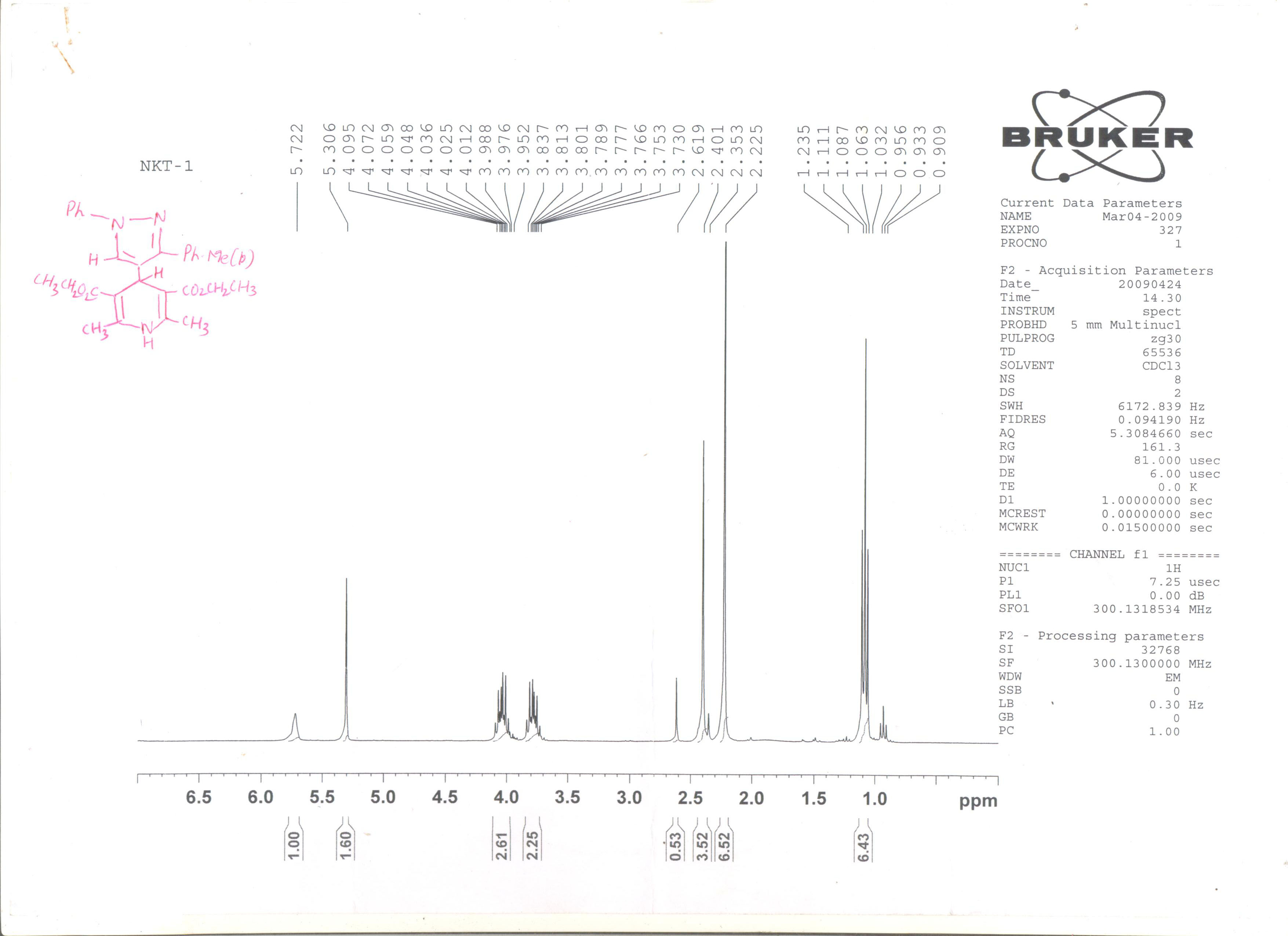

Supplement: Additional file 1 — 1HNMR spectrum of compound 2b. [file 2191-2858-1-5-S1.JPEG]

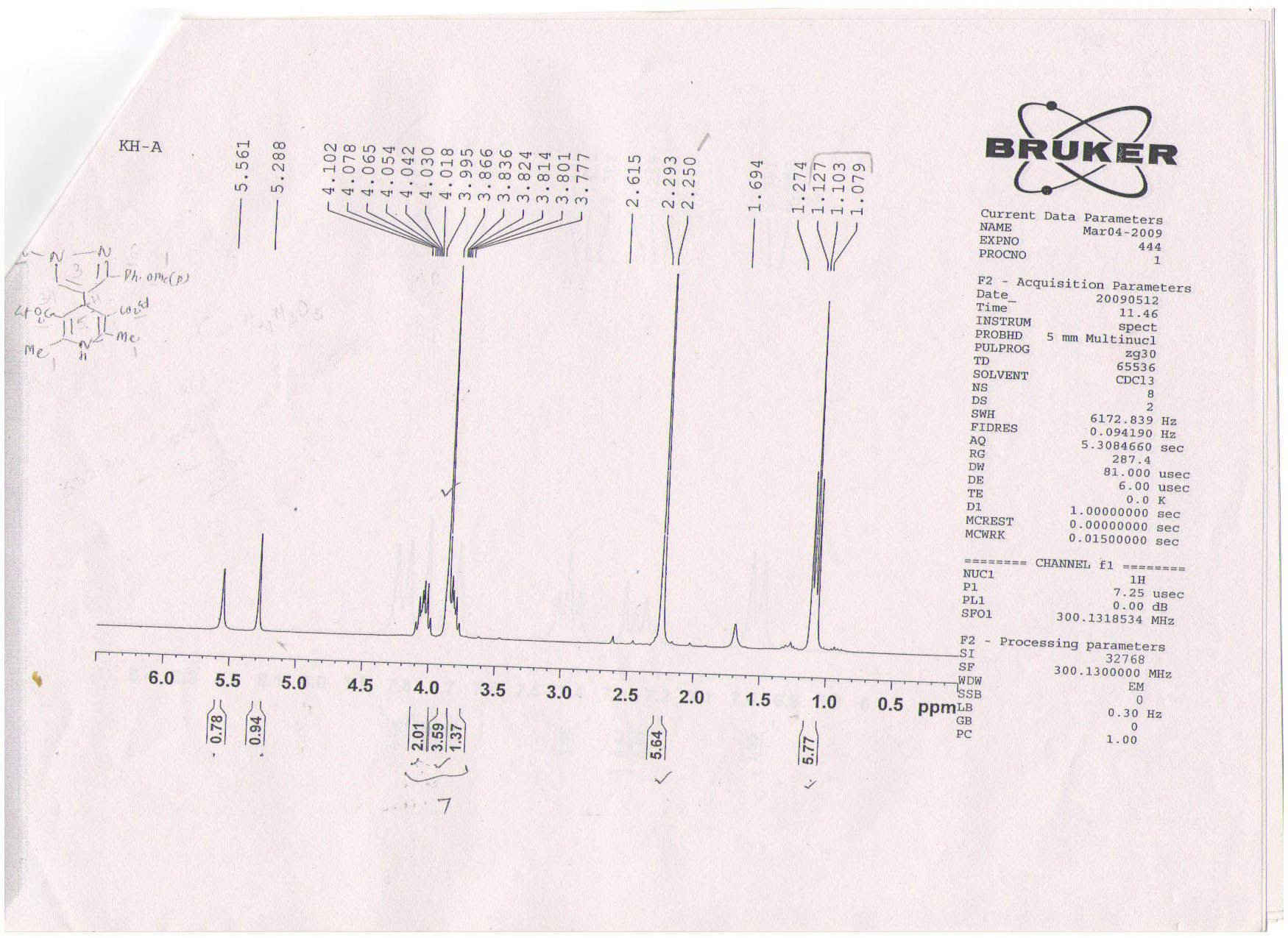

Supplement: Additional file 2 — 1HNMR spectrum of compound 2c. [file 2191-2858-1-5-S2.JPEG]

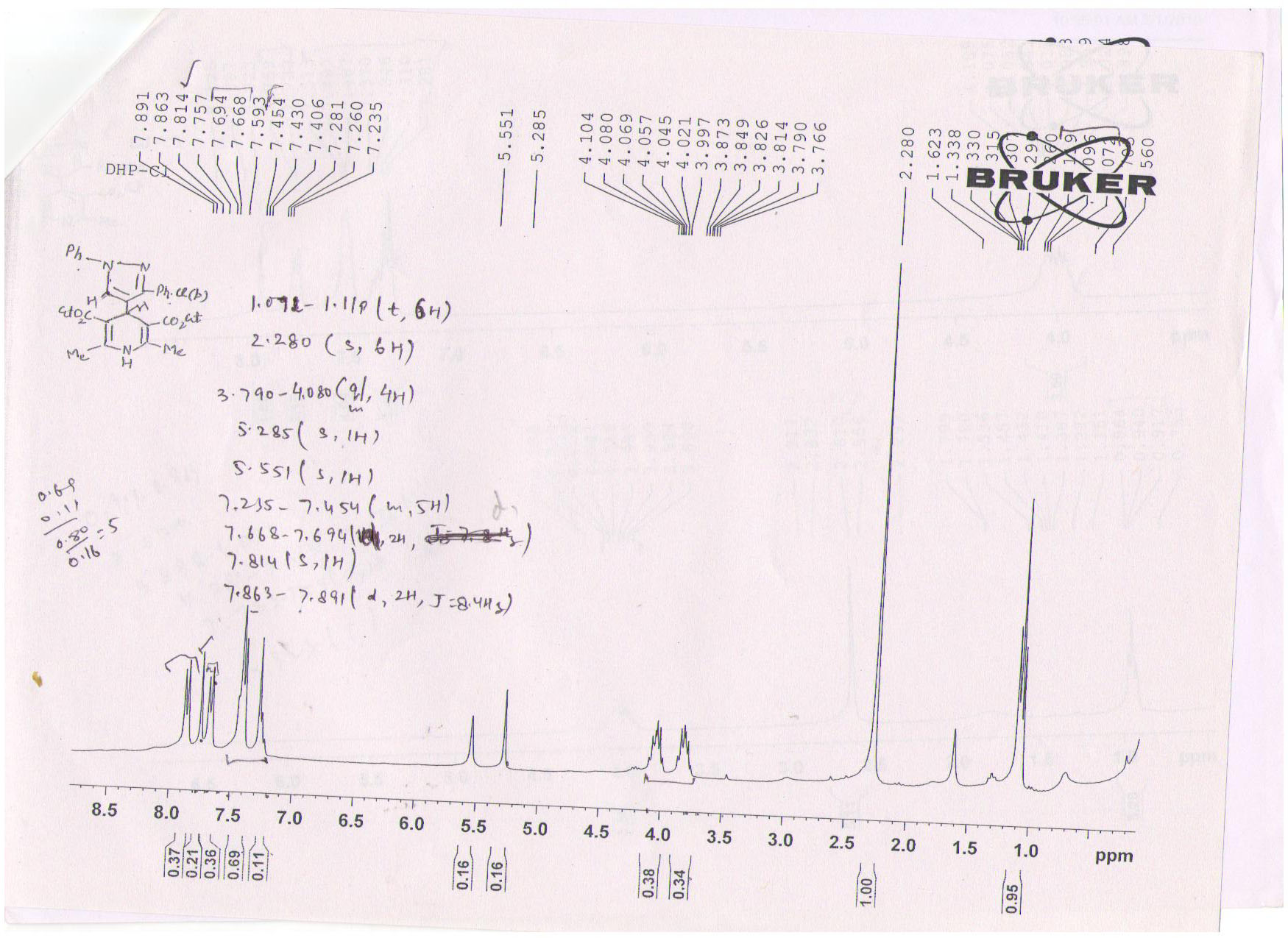

Supplement: Additional file 3 — 1HNMR spectrum of compound 2e. [file 2191-2858-1-5-S3.JPEG]

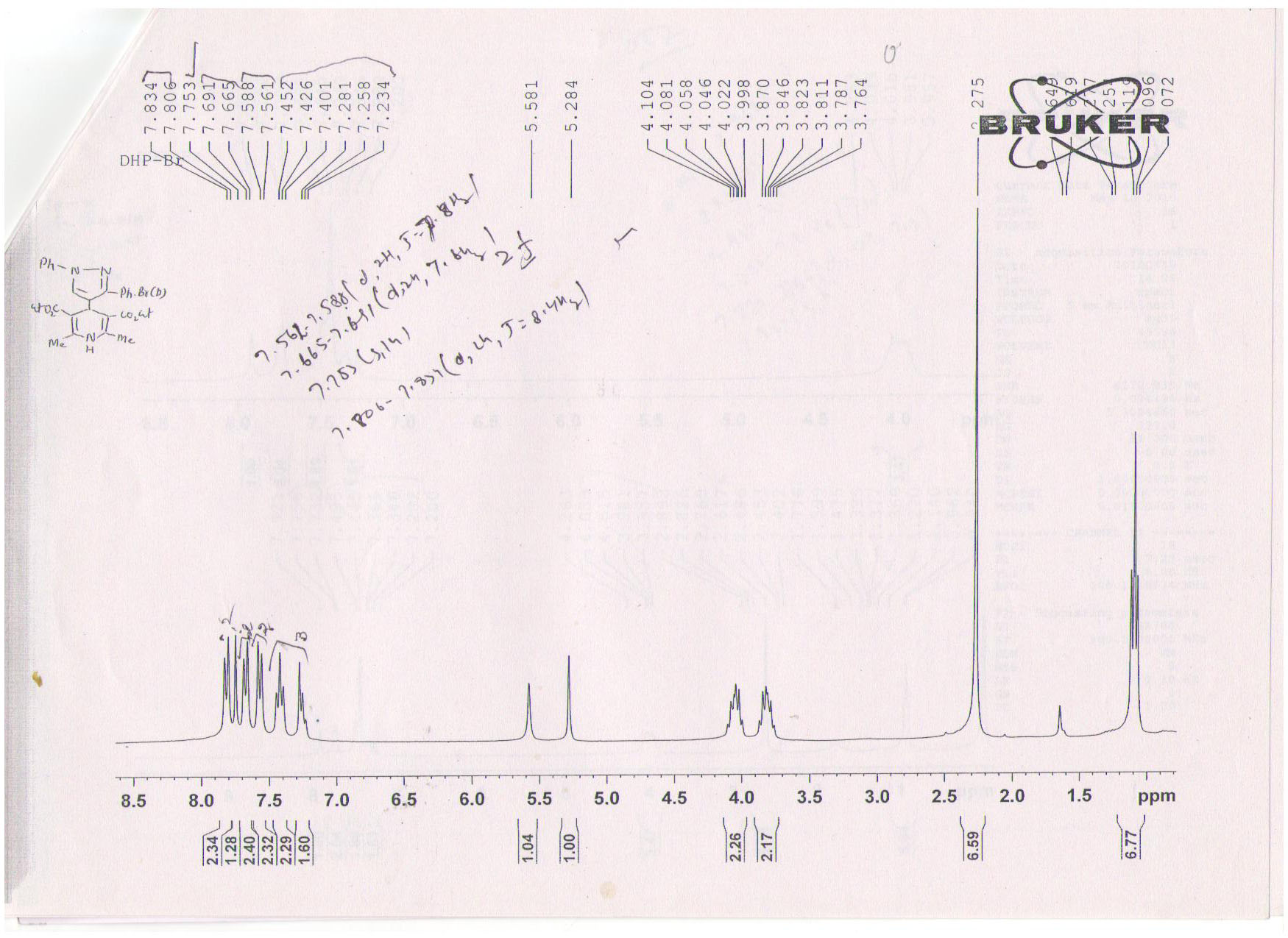

Supplement: Additional file 4 — 1HNMR spectrum of compound 2f. [file 2191-2858-1-5-S4.JPEG]

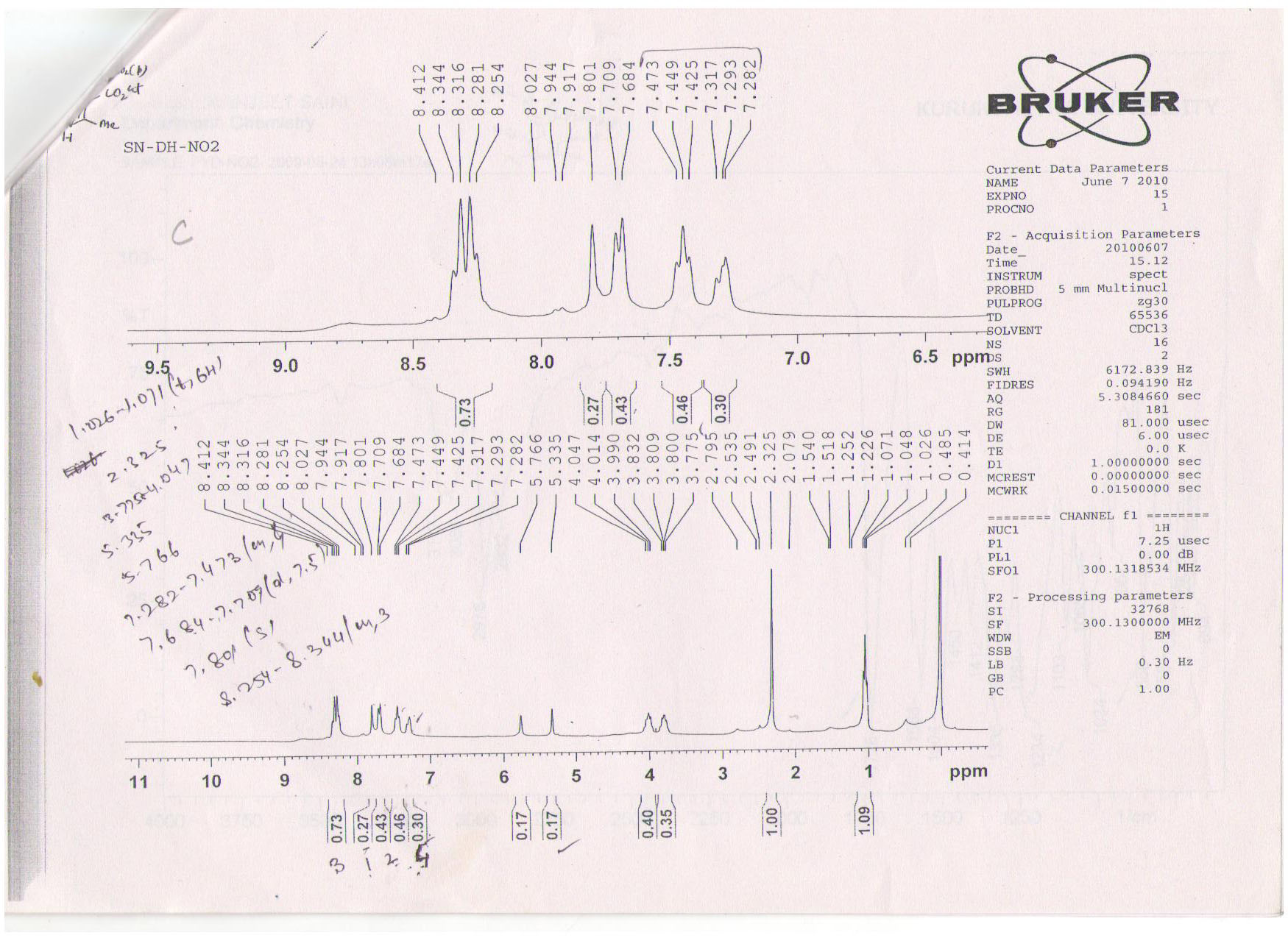

Supplement: Additional file 5 — 1HNMR spectrum of compound 2g. [file 2191-2858-1-5-S5.JPEG]

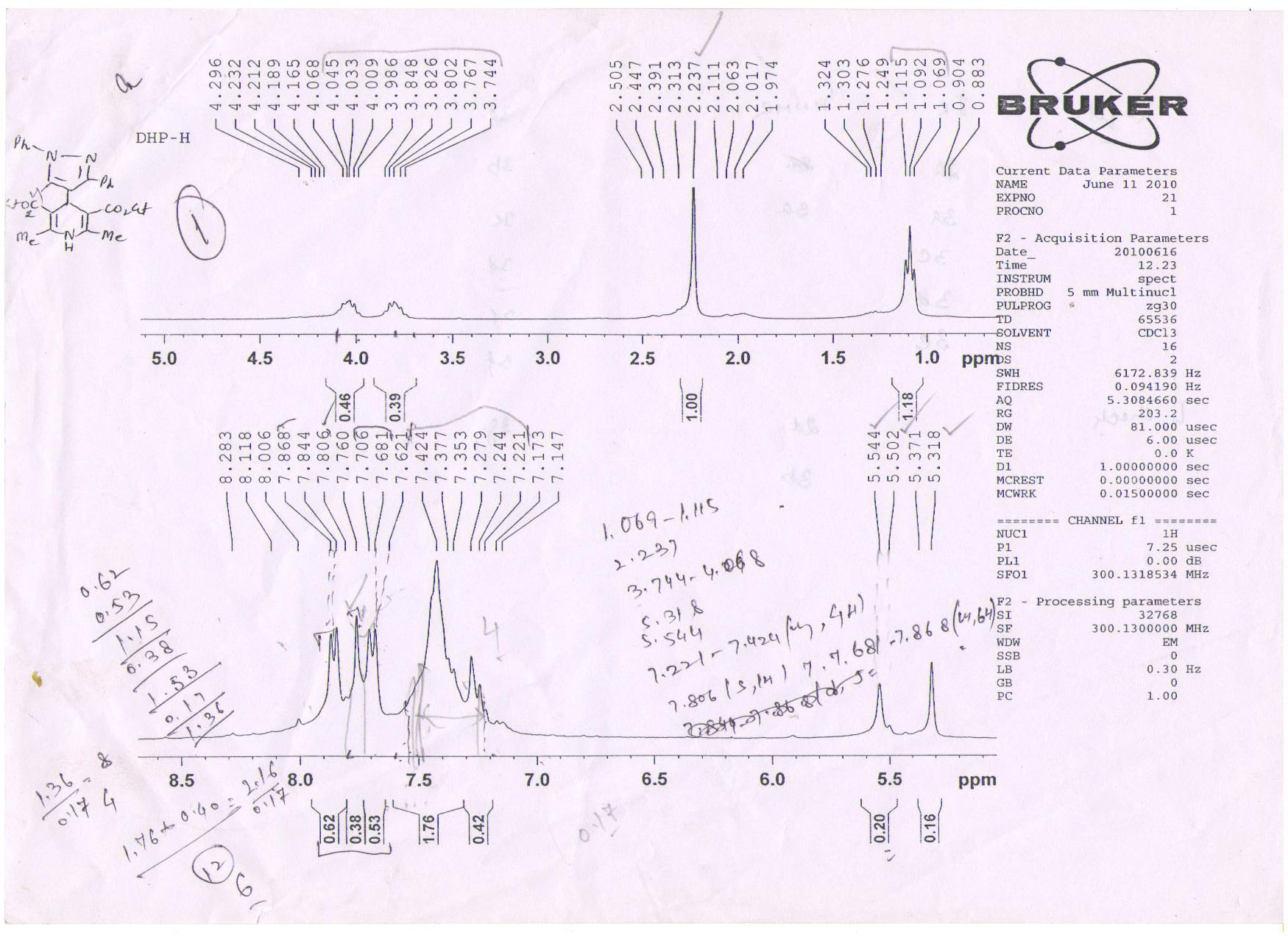

Supplement: Additional file 6 — 1HNMR spectrum of compound 3a. [file 2191-2858-1-5-S6.JPEG]

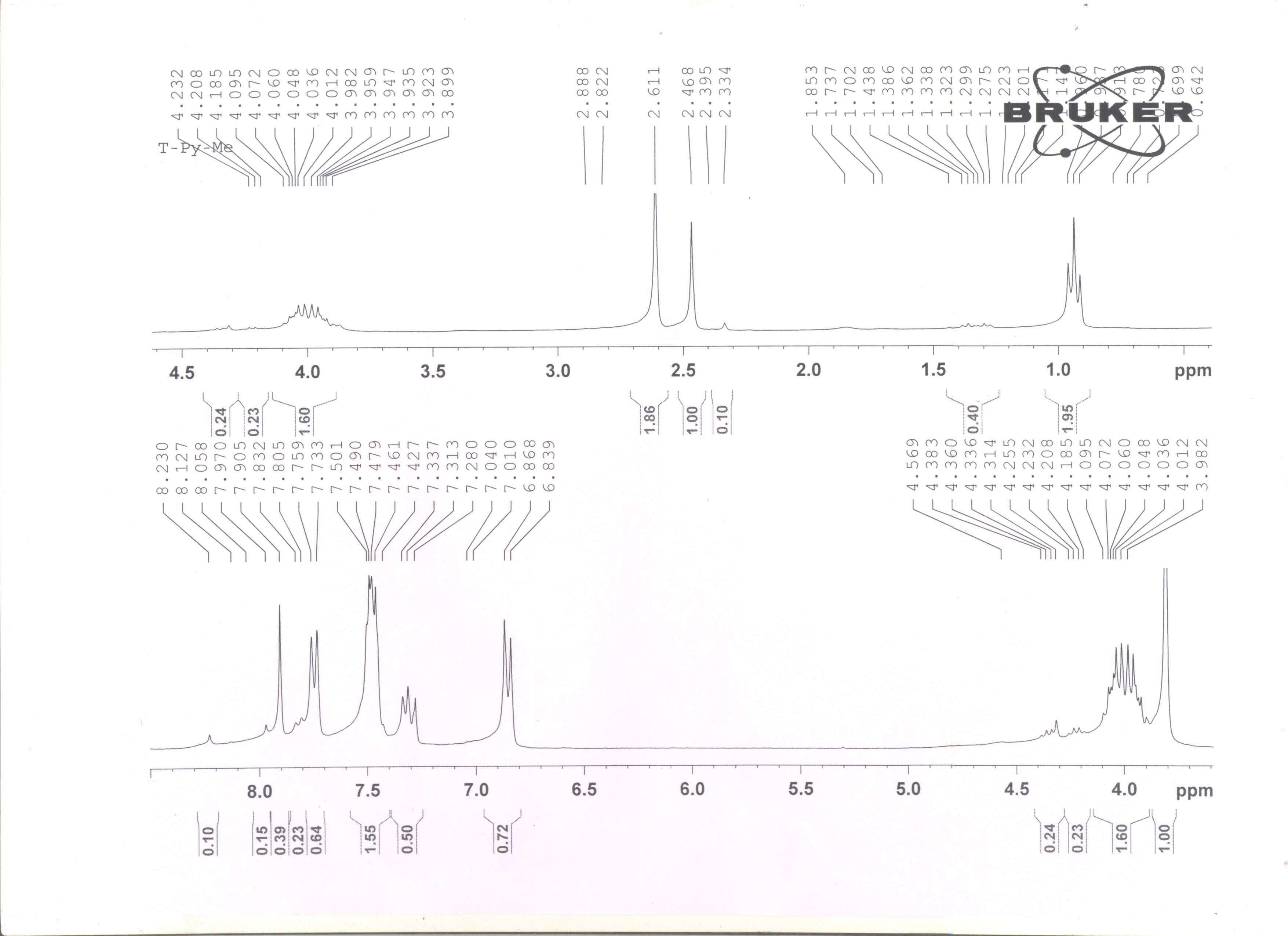

Supplement: Additional file 7 — 1HNMR spectrum of compound 3b. [file 2191-2858-1-5-S7.JPEG]

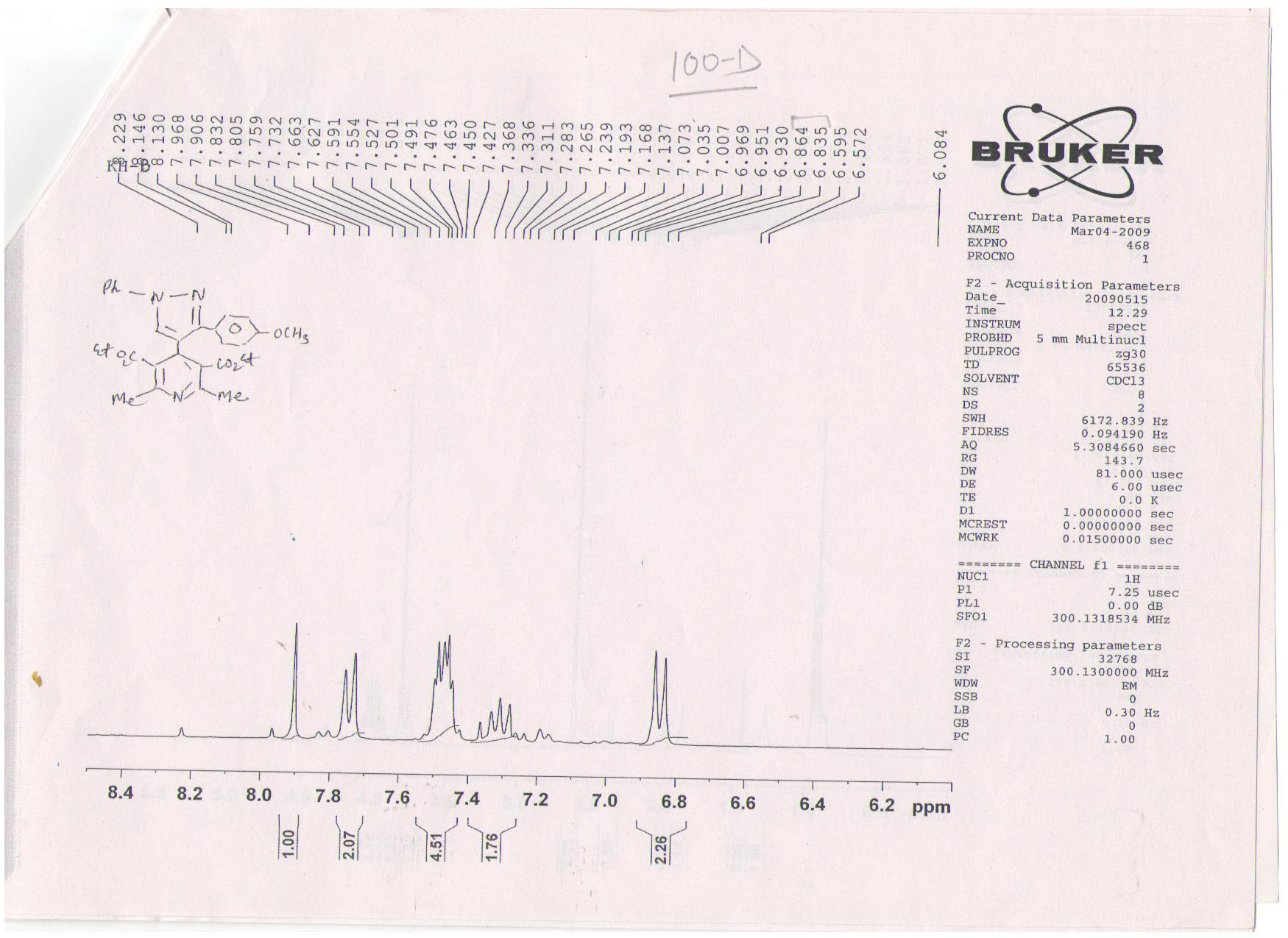

Supplement: Additional file 8 — 1HNMR spectrum of compound 3c. [file 2191-2858-1-5-S8.JPEG]

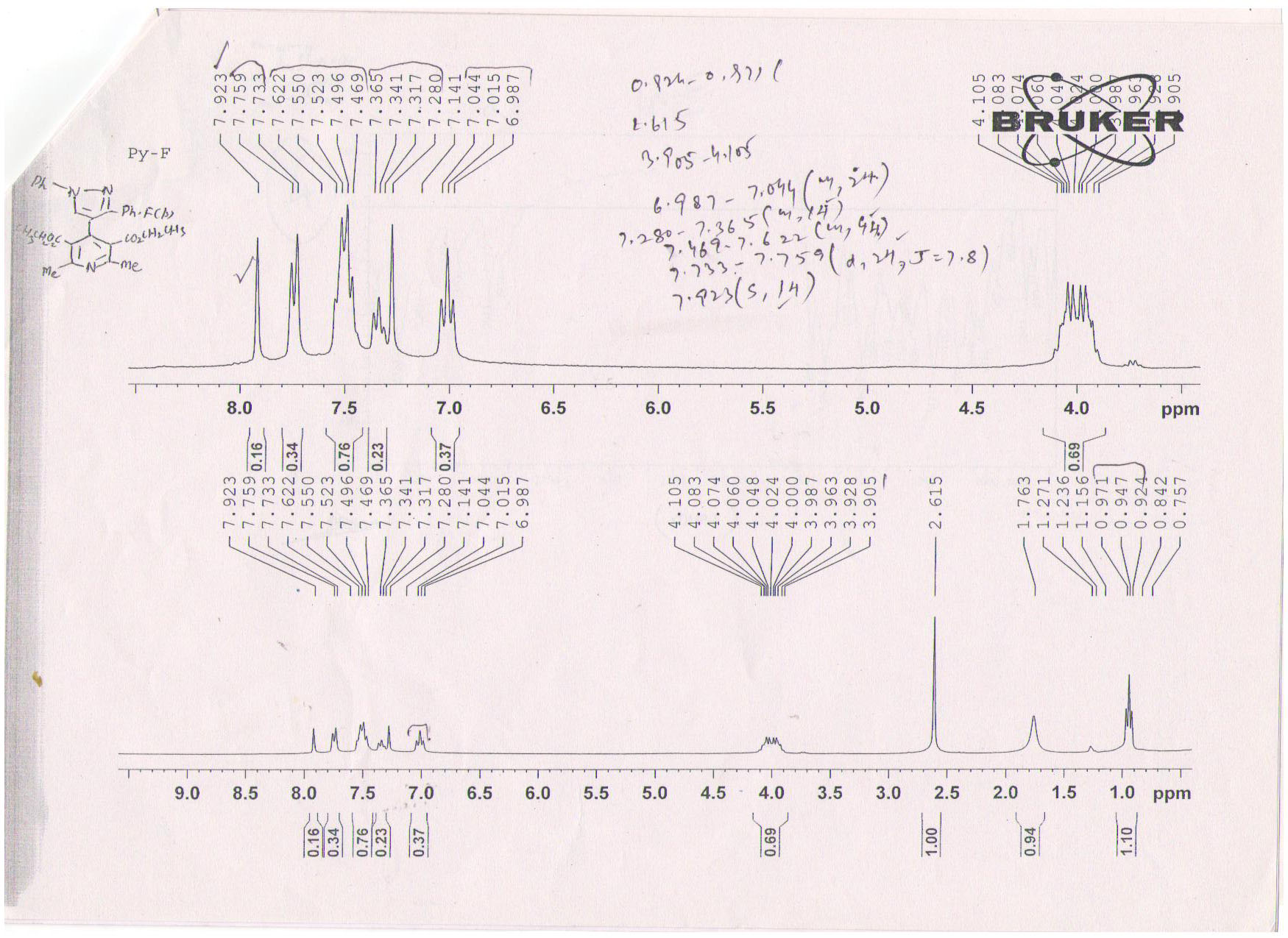

Supplement: Additional file 9 — 1HNMR spectrum of compound 3d. [file 2191-2858-1-5-S9.JPEG]

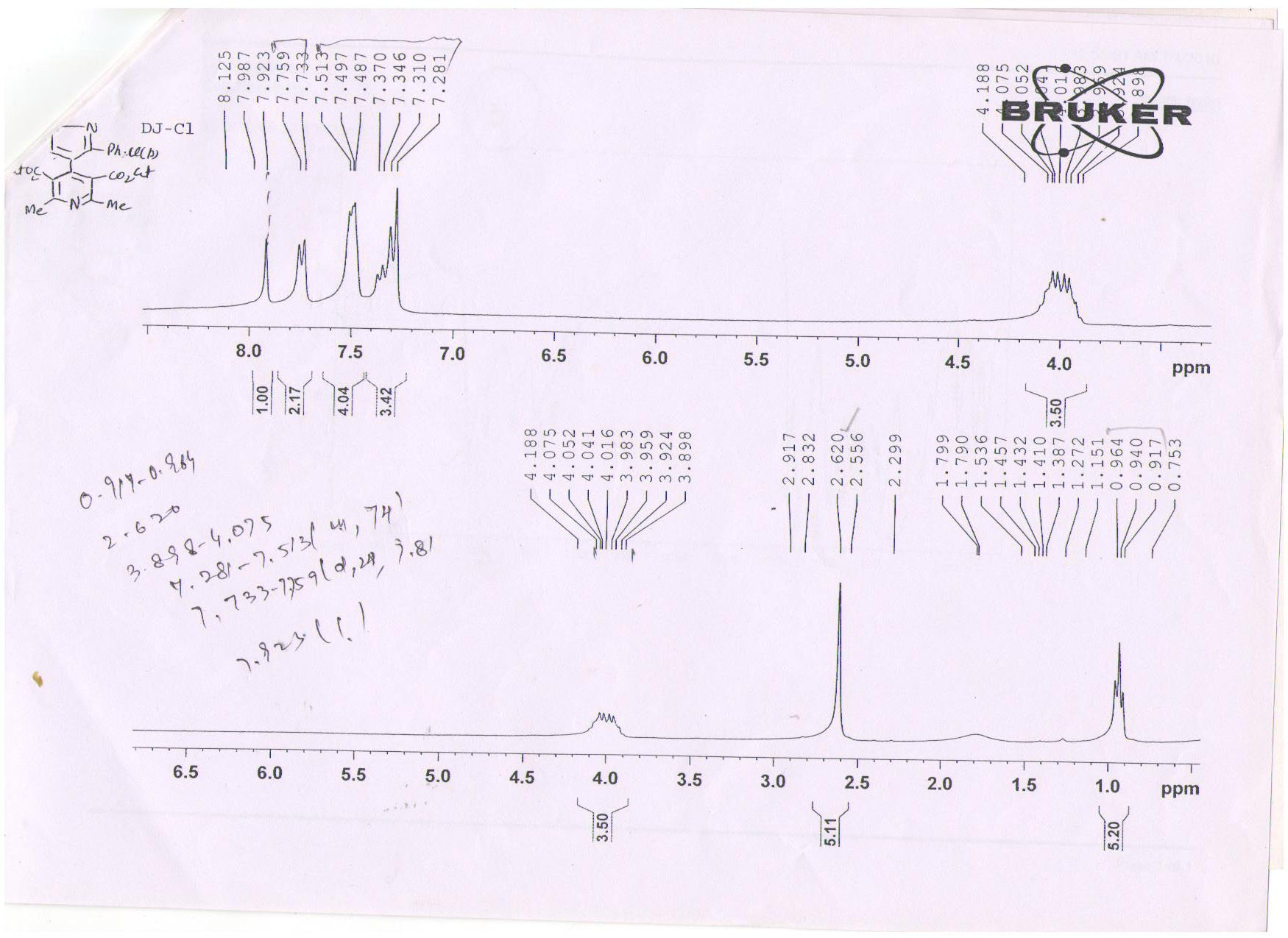

Supplement: Additional file 10 — 1HNMR spectrum of compound 3e. [file 2191-2858-1-5-S10.JPEG]

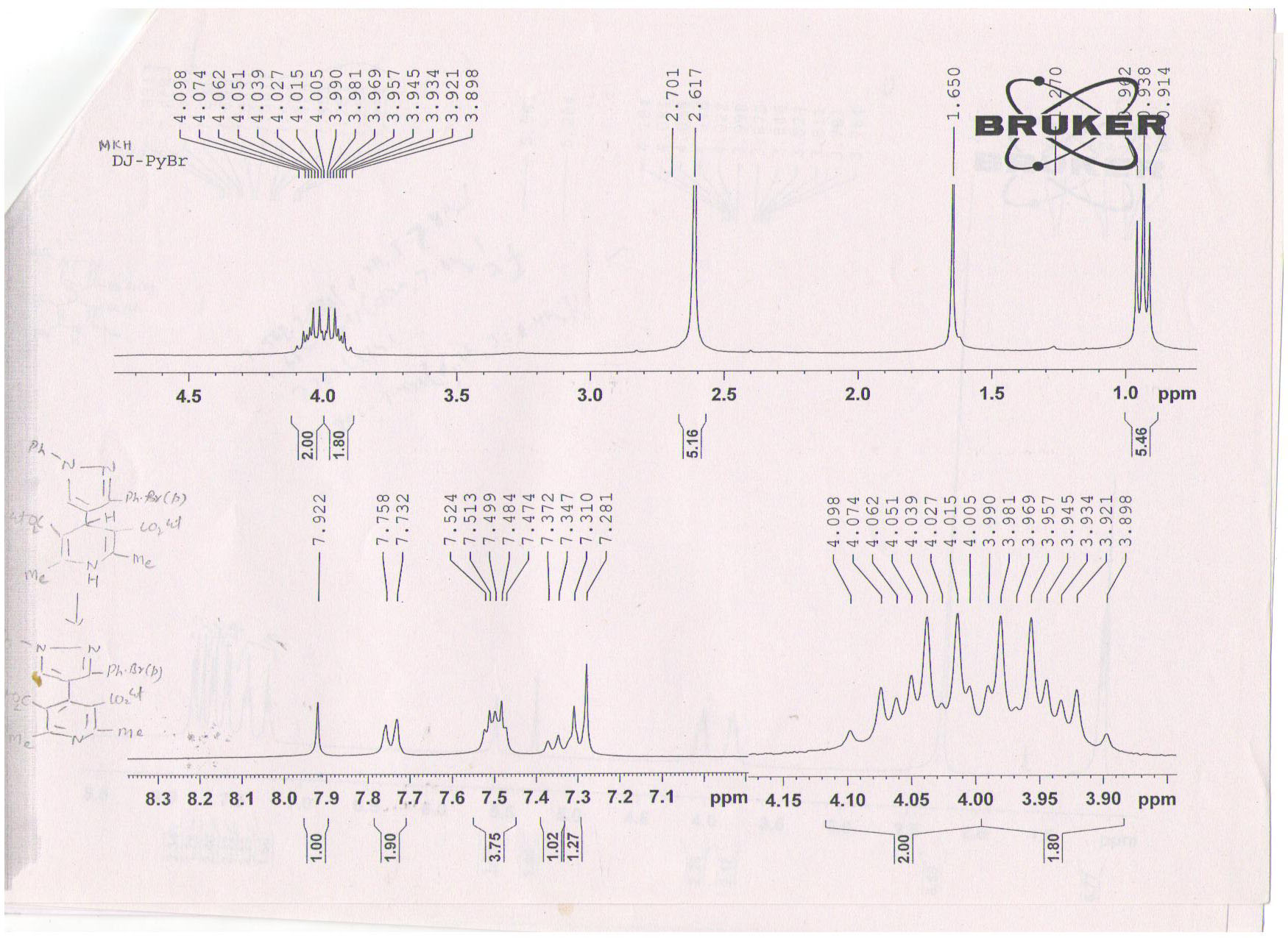

Supplement: Additional file 11 — 1HNMR spectrum of compound 3f. [file 2191-2858-1-5-S11.JPEG]

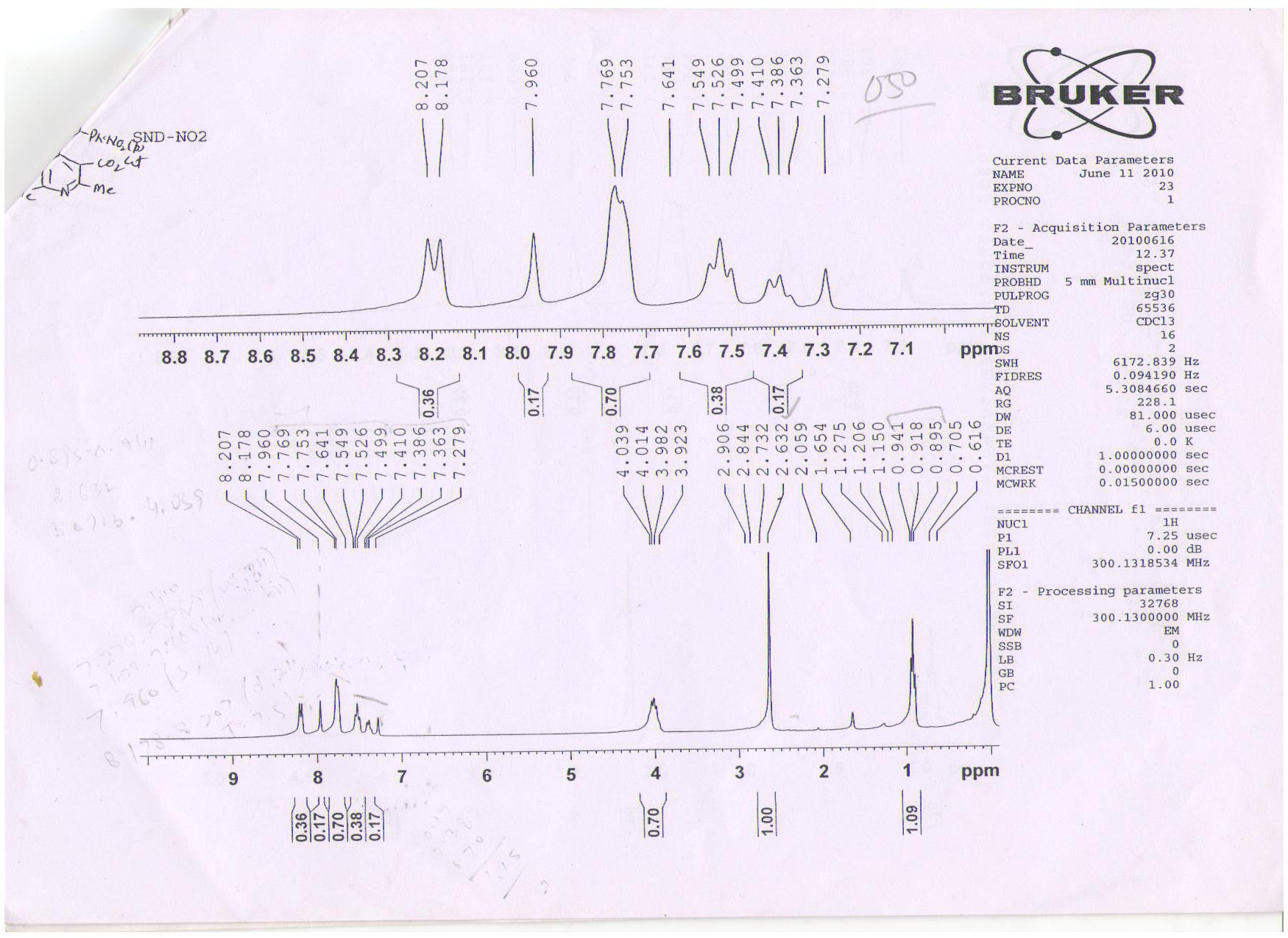

Supplement: Additional file 12 — 1HNMR spectrum of compound 3g. [file 2191-2858-1-5-S12.JPEG]
